# Global disparities in intestinal helminth infection dynamics among food handlers from 1990 to 2022: A call for targeted interventions

**DOI:** 10.1016/j.parepi.2026.e00552

**Published:** 2026-10-09

**Authors:** Behrooz Ahmadi, Meysam Olfatifar, Milad Badri, Alireza Ghorbani, Mahdi Fakhar

**Affiliations:** a Gastroenterology and Liver Diseases Research Center, Research Institute for Gastroenterology and Liver Diseases, Shahid Beheshti University of Medical Sciences, Tehran, Iran; b Gastroenterology and Hepatology Diseases Research Center, Qom University of Medical Sciences, Qom, Iran; c Safety and Health Research Center, Qazvin University of Medical Sciences, Qazvin, Iran; d Department of Pathobiology, Faculty of Veterinary Medicine, Urmia University, Urmia, Iran

**Keywords:** Force of infection, Infection-clearance rate, Intestinal helminth infections, Food handlers

## Abstract

Intestinal helminths infect over 1.5 billion people globally, with food handlers serving as critical vectors in their transmission. This study aimed to quantify the transmission dynamics of intestinal helminths among food handlers by estimating the Force of Infection (FoI) and Infection Clearance Rate (ICR), providing evidence for targeted public health interventions. We employed a Susceptible-Infected-Susceptible (SIS) compartmental model, formalized by ordinary differential equations (ODEs), to estimate FoI and ICR. Crucially, these parameters were inferred from cross-sectional prevalence data rather than longitudinal individual follow-up. Models were calibrated using data from a global meta-analysis comprising 220,705 food handlers across 29 countries. We assessed variations in transmission dynamics across regions, parasite species, demographics, sample sources, environments, and diagnostic methods. Parameter optimization was performed using a differential evolution algorithm to ensure robustness. Global FoI and ICR estimates were 0.0050 and 0.0087, respectively. Since the clearance rate exceeds the force of infection, the model indicates a net decline in the prevalence of infection. Declining trends predominated across most strata, including all three regions, most demographic subgroups, and 3 of 10 parasite species. However, India and Kenya showed expanding burdens. Regionally, Asia and South America had higher transmission parameters than Africa. Nationally, Iran exhibited the highest FoI and ICR (declining trend), while Kenya showed the lowest point estimates. *Trichostrongylus* spp. showed the highest species-level parameters; *E. vermicularis* the lowest. Elevated values were observed in males, the 26–30 age group, high-HDI countries (with declining trend due to effective surveillance), illiterate individuals, and shops/restaurants. This study provides the first global estimates of FoI and ICR for intestinal helminths among food handlers, revealing significant regional, species-related, and demographic differences. These findings highlight the need for targeted public health interventions including mass drug administration (MDA), hygiene education, improved diagnostics, and stricter regulatory measures—in strata with elevated transmission pressure.

## 1 Introduction

More than half of the world's population is impacted by helminthic infections, especially soil-transmitted helminths (STHs), with more than 1.5 billion people affected (Agrawalet al., 2024). These infections, such as *Ascaris lumbricoides*, hookworm, and *Trichuris trichiura*, have a profound impact on health, education, nutrition, and economic development (Wakid and Al-Refai, 2024). In 2021, the global burden of intestinal nematode infections, which include STHs, was estimated at 1.38 million DALYs (95% UI: 0.92–2.02) (Zheng et al., 2026). These infections are most prevalent in tropical and subtropical climates, where the physical and social conditions support the survival and transmission of STHs (Freeman et al., 2019; Sturrock et al., 2017).

Controlling STHs among food handlers represents a critical public health leverage point, as this group acts as a key reservoir for community transmission through food contamination (Mardu et al., 2020; Tappes et al., 2019). Consequently, interventions like regular parasitological screening, deworming programs, and health education are essential to disrupt transmission cycles (Garn et al., 2022; Nath et al., 2020). However, their success in endemic settings remains suboptimal due to two interconnected epidemiological drivers: persistently high force of infection (FoI) (Kaslow, 2021; Lydeamore et al., 2020), driven by occupational exposure to contaminated soil, water, and produce during food handling (Agrawal et al., 2024; Zaglool et al., 2011), and chronically low infection clearance rate (ICR), resulting from rapid reinfection and anthelmintic limitations. Reducing FoI through improved sanitation, personal hygiene, and environmental interventions minimizes exposure risk, while enhancing ICR through timely treatment shortens the duration individuals remain infectious. Together, these parameters determine the equilibrium of infection and are fundamental to sustainable control strategies.

Accurately quantifying the FoI and ICR among food handlers is crucial for interrupting transmission and filling a key knowledge gap in occupational helminth epidemiology. Although prevalence studies are plentiful, reliable dynamic metrics like FoI and ICR, which are essential for anticipating the impact of interventions, remain largely unreported. This study aims to address a critical gap by generating global and subgroup-level estimates (e.g., by occupation type and region) using a novel analytical framework. We defined “high-risk” populations based on a fundamental dynamic rule in infectious disease modeling: a population is considered high-risk when the FoI exceeds the Infection ICR, leading to a net increase in infection prevalence. Our objectives are to: (1) evaluate the FoI and ICR of intestinal helminths among food handlers; (2) identify high-risk subgroups based on this dynamic threshold; and (3) inform targeted interventions—including precision deworming schedules, food safety protocols, Human Development Index (HDI)-specific strategies, and environmental modifications to interrupt transmission and reduce the public health burden associated with these infections.

## 2 Methods

### 2.1 Data source and analytical approach

To calibrate our transmission models and estimate the FoI and ICR, we leveraged data from a recent global meta-analysis on intestinal helminth infections among food handlers from 1990 to 2022 (Eslahi et al., 2023). This comprehensive dataset encompasses 127 independent studies spanning 29 countries, representing 220,705 screened food handlers across diverse geographic and institutional settings. Wherein we applied random-effects meta-analytical methods stratified across predefined demographic, geographic, and temporal subgroups. This reanalysis generated annualized, subgroup-specific prevalence estimates that served as calibration targets for our mathematical models.

Thus, the present study constitutes a retrospective analysis of surveillance data spanning 32 years (1990–2022), which were employed to calibrate a dynamic susceptible-infected-susceptible (SIS) model. Given the scarcity of individual-level longitudinal data pertaining to global food handler populations, this approach enables the inference of transmission and clearance parameters from observed temporal trends in infection burden, providing a robust alternative to static cross-sectional analyses predicated upon the assumption of immediate endemic equilibrium. To ensure robust parameter estimation and prevent overfitting, we restricted our modeling framework exclusively to geographic and subgroup units containing ≥3 temporally distinct annual prevalence measurements, thereby maintaining statistical reliability throughout the inference process. Applying this criterion, 35 out of 55 initial subgroups met the threshold for temporal resolution, despite all 10 helminth species meeting the criteria. These selected units are reported in Table 1, Table 2. This filtering ensured that all presented FoI and ICR estimates were derived from statistically robust model fits, avoiding the high uncertainty associated with under-determined systems (i.e., fewer than three data points for two parameters).

**Table 1:** Force of Infection (FoI) and Infection Clearance Rate (ICR) with 95% Confidence Intervals (CI) and Root Mean Square Error (RMSE) for Intestinal Helminthic Infections among Food Handlers, by Global and Country Groups⁎.

Groups	FoI	FoI Lower	FoI Upper	ICR	ICR Upper	ICR Upper	RSME
Global	0.005061	0.005055	0.005369	0.008758	0.008753	0.009020	0.0787
Africa	0.0105	0.0101	0.0136	0.0161	0.0158	0.0184	0.1272
Asia	0.0919	0.0856	0.1340	0.1301	0.1261	0.1535	0.1170
South America	0.1238	0.0985	0.2275	0.1667	0.1473	0.2176	0.0832
Brazil	0.1122	0.0399	0.2117	0.1499	0.0894	0.2191	0.0471
Ethiopia	0.0689	0.0660	0.0874	0.0801	0.0787	0.0880	0.0889
India	0.2953	0.2263	0.7024	0.1302	0.0657	0.4987	0.0721
Iran	0.4235	0.2587	0.6111	0.5116	0.3792	0.6009	0.0358
Kenya	0.0268	0.0128	0.0470	0.0127	0.0000	0.0310	0.0803
Nigeria	0.0963	0.0874	0.1164	0.1113	0.1069	0.1202	0.0773
Saudi Arabia	0.0420	0.0315	0.1171	0.0784	0.0687	0.1403	0.1083

⁎ Note: Values are rounded to four or five decimal places depending on the magnitude of the estimate to preserve the distinction between point estimates and confidence intervals.

**Table 2:** Force of Infection (FoI) and Infection Clearance Rate (ICR) with 95% Confidence Intervals (CI) and Root Mean Square Error (RMSE) for Different Species of Intestinal Helminthic Parasites among Food Handlers⁎.

Groups	FoI	FoI Lower	FoI Upper	ICR	ICR Lower	ICR Upper	RSME
*Ascaris lumbricoides*	0.0298	0.0295	0.0402	0.025	0.021	0.0301	0.0392
*Enterobius vermicularis*	0.0014	0.0009	0.0025	0.0005	0.0000	0.0016	0.0229
Hookworm	0.00781	0.00779	0.01073	0.01494	0.01492	0.01771	0.0406
*Hymenolepis nana*	0.000161	0.000152	0.000341	0.000136	0.000049	0.000192	0.0116
*Strongyloides stercoralis*	0.0727	0.0682	0.1442	0.0601	0.0029	0.0771	0.0182
*Schistosoma mansoni*	0.1550	0.1426	0.2974	0.0835	0.0711	0.2225	0.0099
*Trichuris trichiura*	0.0055	0.0052	0.0094	0.0109	0.0107	0.0148	0.0261
*Taenia saginata*	0.1321	0.1255	0.2670	0.0599	0.0230	0.1584	0.0299
*Taenia* spp.	0.1384	0.1296	0.2749	0.0499	0.0413	0.1801	0.0150
*Trichostrongylus* spp.	0.5925	0.0000	0.8875	0.6484	0.0734	0.9272	0.0066

⁎ Note: Values are rounded to four or five decimal places depending on the magnitude of the estimate to preserve the distinction between point estimates and confidence intervals.

### 2.2 Subgroup stratification

Subgroup analyses were conducted to assess differences in FoI and ICR across various demographic, geographic, and methodological categories, HDI, including region, parasite species, sex, age, education levels, and diagnostic techniques.

### 2.3 Compartmental modeling approach

To estimate the FoI and ICR, we implemented a compartmental SIS model formalized by the following system of ordinary differential equations (ODEs). The SIS framework was selected based on the immunological profile of intestinal helminth infections, which fail to confer lasting protective immunity permitting immediate reinfection post-clearance. The transmission dynamics are governed by:

$$\frac{\mathrm{ds}}{t}=-\lambda \times S\times I+\rho \times I$$

$$\;\frac{\mathrm{dI}}{t}=\lambda \times S\times I-\rho \times I$$

In these equations: S and I represent the proportions of susceptible and infected individuals at time t, respectively. The parameter λ represents the FoI reflecting the rate at which susceptible individuals acquire the infection. The parameter ρ denotes the ICR, which captures the rate at which infections are resolved through host immunity, treatment, or other mechanisms. In this framework, the dynamics of infection prevalence are governed by the interplay between the FoI and the ICR. This relationship reflects the basic reproductive number (R_0_ = FOI/ICR), serving as a key indicator of transmission potential. Specifically, FoI represents the rate at which susceptible individuals acquire infection, while ICR denotes the rate at which infections are resolved. Consequently, an increasing trend in prevalence occurs when FoI exceeds ICR, indicating that new infections outpace recoveries (R_0>1_). Conversely, a declining trend is observed when ICR exceeds FoI, signifying that recovery rates surpass acquisition rates (R_0<1_). This balance between acquisition and clearance determines whether the infection burden is expanding or contracting within the population.

### 2.4 Model calibration

To estimate the FoI and ICR across global, regional, national, and subgroup levels, we constructed dedicated computational models for each distinct population stratum. These models were rigorously calibrated by fitting to empirical annual prevalence data specific to each category. Parameter optimization was performed using the differential evolution algorithm, implemented via the DEoptim package in R (Mullen et al., 2011), selected for its robustness in handling non-linear, discontinuous systems and inherent insensitivity to initial parameter values, thereby ensuring reliable convergence. To ensure biological plausibility, we constrained the search space for both FoI and ICR to the interval [0,1], as these parameters represent rates per time unit. Convergence was rigorously assessed by inspecting optimization messages; only fits with successful convergence were retained. Model fit quality was assessed using the Root Mean Square Error (RMSE), chosen for its direct interpretability in prevalence units (0–1) and suitability for non-linear systems. Uncertainty was quantified via bootstrap resampling (95% CI), ensuring all estimates remained within biologically valid bounds.

## 3 Results

### 3.1 FoI and ICR at global, regional and national levels

Globally, the estimated FoI and ICR for intestinal helminths among food handlers were 0.005061 (95% CI: 0.005055–0.005369) and 0.008758 (95% CI: 0.008753–0.009020), respectively. Notably, the ICR exceeded the FoI (ICR > FoI), indicating a net reduction in the number of infected individuals. This dynamic suggests that, under current conditions, the infection burden is in a state of decline as the rate of clearance outpaces the rate of new infections. At the regional level, the estimates varied considerably. In Africa, FoI was 0.0105 (95% CI: 0.0101–0.0136), and ICR was 0.0161 (95% CI: 0.0158–0.0184). In Asia, noticeably higher values were observed, with FoI at 0.0919 (95% CI: 0.0856–0.1340) and ICR at 0.1301 (95% CI: 0.1261–0.1535). South America region (aggregated from studies in Brazil, Paraguay, and Peru) also showed elevated rates, with FoI at 0.1238 (95% CI: 0.0985–0.2275) and ICR at 0.1667 (95% CI: 0.1473–0.2176). Across regions, the infection burden demonstrates a declining trend (ICR > FoI), with South America exhibiting the highest transmission intensity. At the national level, among the countries meeting the temporal resolution criteria, the highest FoI and ICR were observed in Iran, estimated at 0.4235 (95% CI: 0.2587–0.6111) and 0.5116 (95% CI: 0.3792–0.6009), respectively. In contrast, the lowest values were found in Kenya, with FoI estimated at 0.0268 (95% CI: 0.0128–0.0470) and ICR at 0.0127 (95% CI: 0.0000–0.0310) (Table 1, Figs. 1 and 2). Considering the biological trad off between FOI and ICR, while most national units exhibit a declining trend (ICR > FoI), Kenya and India demonstrate an increasing transmission dynamic. Morover, the model demonstrated robust performance across most subgroups, as indicated by RMSE values. Table 1 shows that national estimates generally had lower RMSEs (range: 0.0358–0.1083) compared to regional estimates (range: 0.0832–0.1272).

**Fig. 1:**
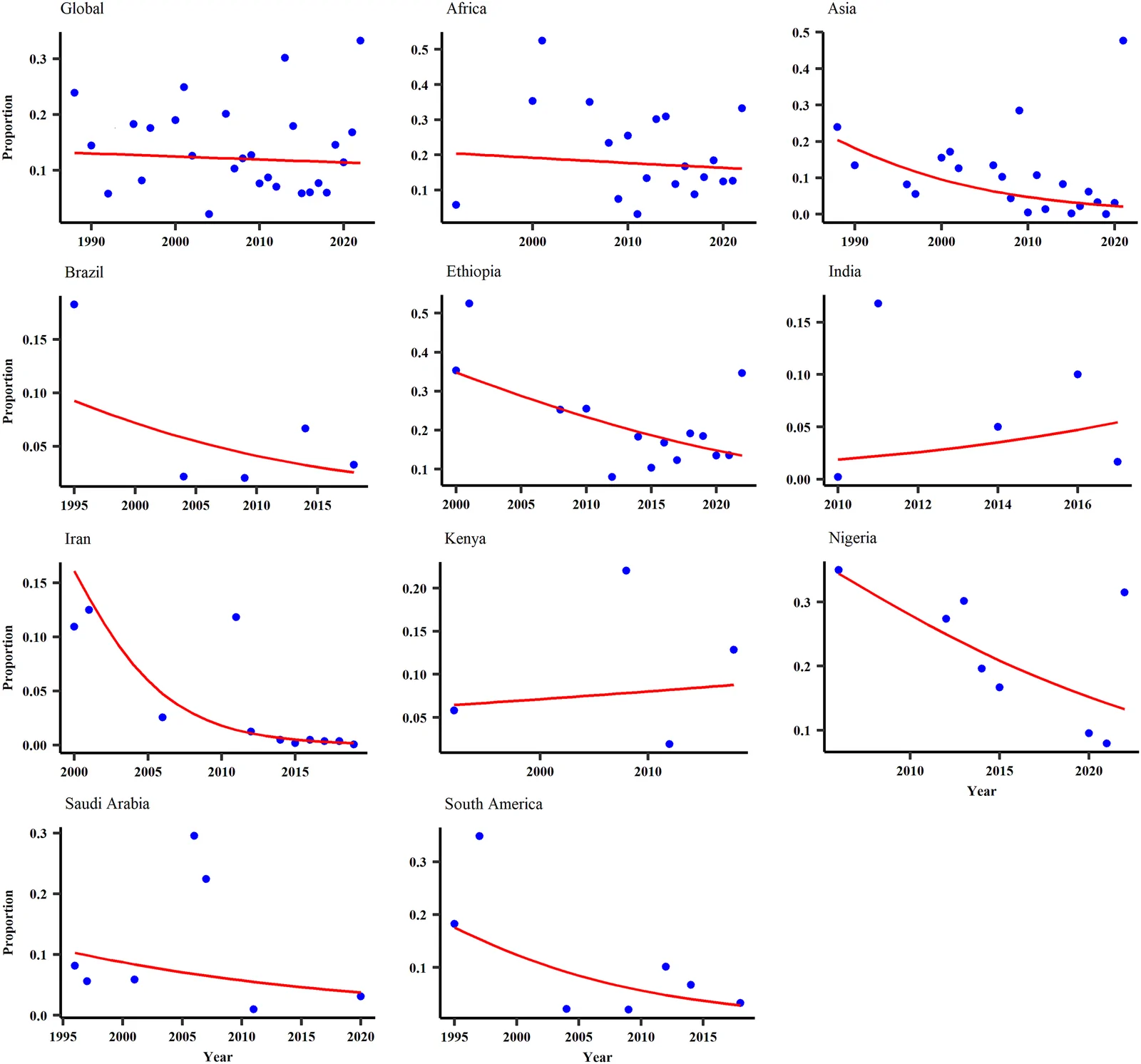
Force of Infection (FoI) and Infection Clearance Rate (ICR) for Intestinal Helminthic Infections among Food Handlers at Global and Country Levels, displaying observed values (blue dots) and fitted values (red lines). (For interpretation of the references to colour in this figure legend, the reader is referred to the web version of this article.)

**Fig. 2:**
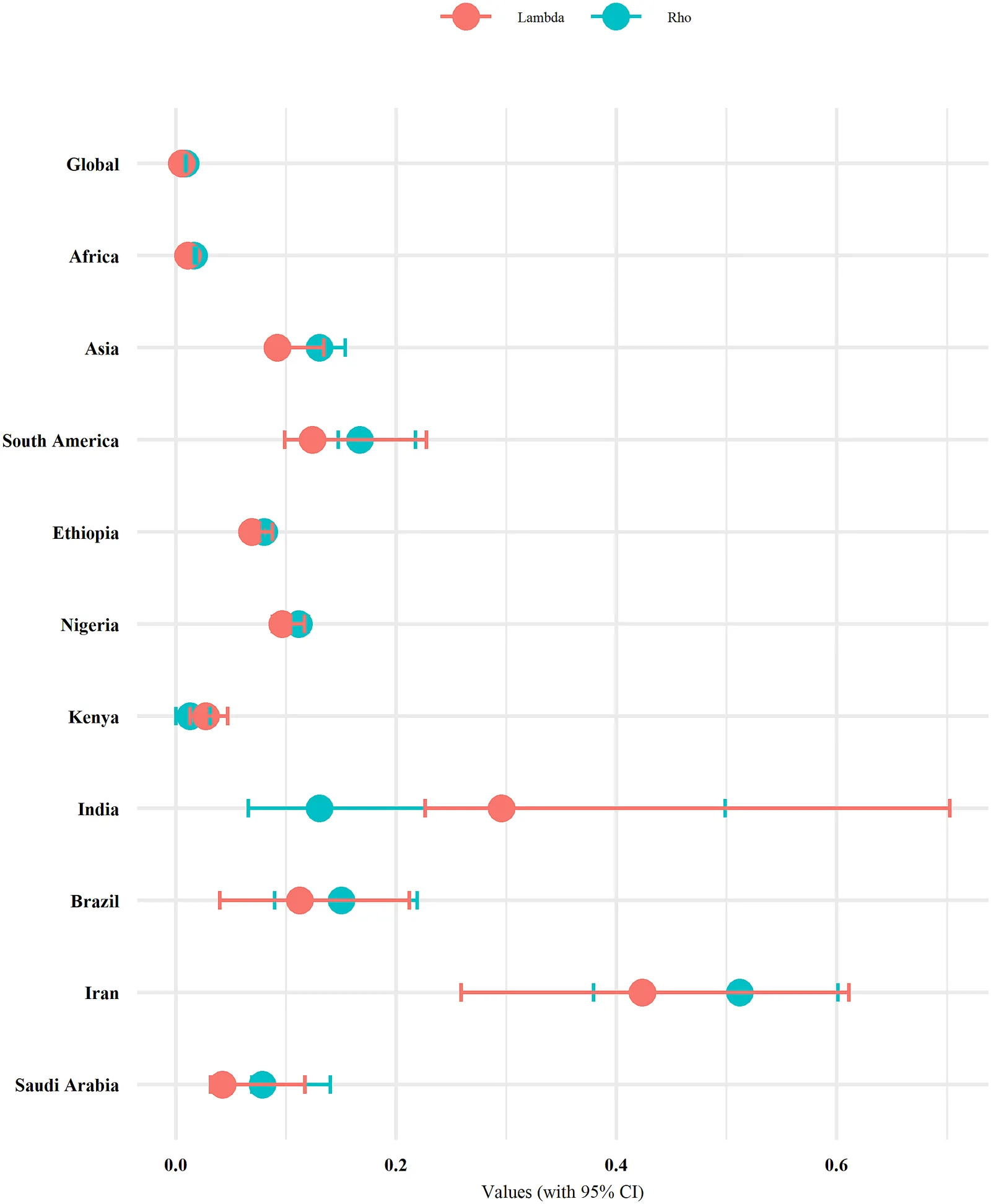
Estimated values (circles) and 95% Confidence Intervals (error bars) for the Force of Infection (FoI) and Infection Clearance Rate (ICR) for Intestinal Helminthic Infections among Food Handlers at Global and Country Levels.

### 3.2 FoI and ICR by intestinal helminth species

The estimated FoI and ICR for different species of intestinal helminths among food handlers varied substantially. The highest values were observed for *Trichostrongylus* spp., with an FoI of 0.5925 (0.0000–0.8875) and an ICR of 0.6484 (0.0734–0.9272). In contrast, the lowest estimates were recorded for *Enterobius vermicularis*, with an FoI of 0.0014 (0.0009–0.0025) and an ICR of 0.0005 (0.0000–0.0016) (Table 2, Figs. 3 and 4). Only three species—hookworm, Trichuris, and Trichostrongylus—exhibited a declining trend. The remaining seven species all displayed increasing trend. The model yielded a good fit for the majority of parasite species, with RMSEs ranging from 0.0066 (Trichostrongylus spp.) to 0.0406 (hookworm) (See Table 2).

**Fig. 3:**
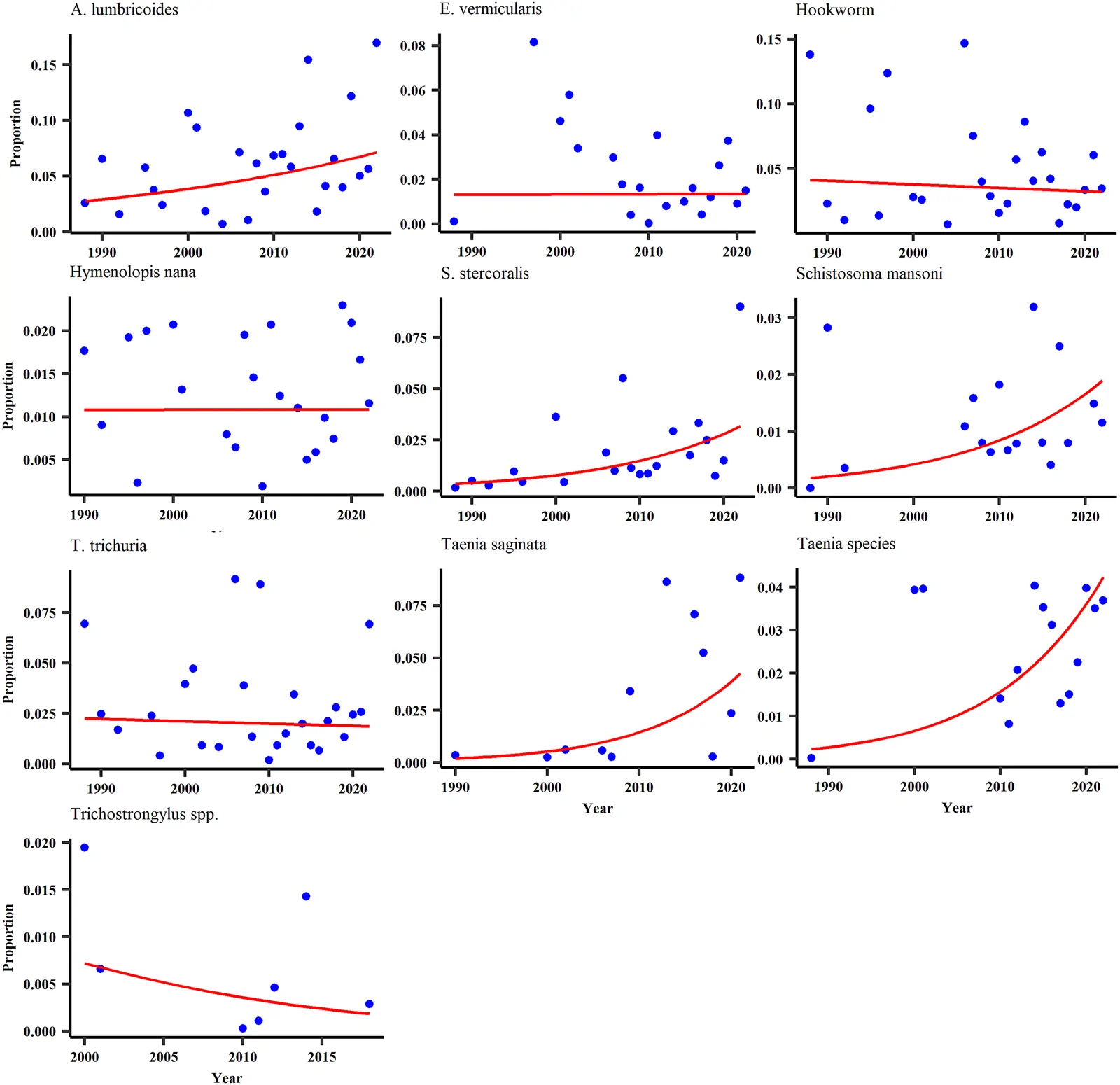
Force of Infection (FoI) and Infection Clearance Rate (ICR) with 95% Confidence Intervals (CI) for Different Species of Intestinal Helminthic Parasites among Food Handlers, displaying observed values (blue dots) and fitted values (red lines). (For interpretation of the references to colour in this figure legend, the reader is referred to the web version of this article.)

**Fig. 4:**
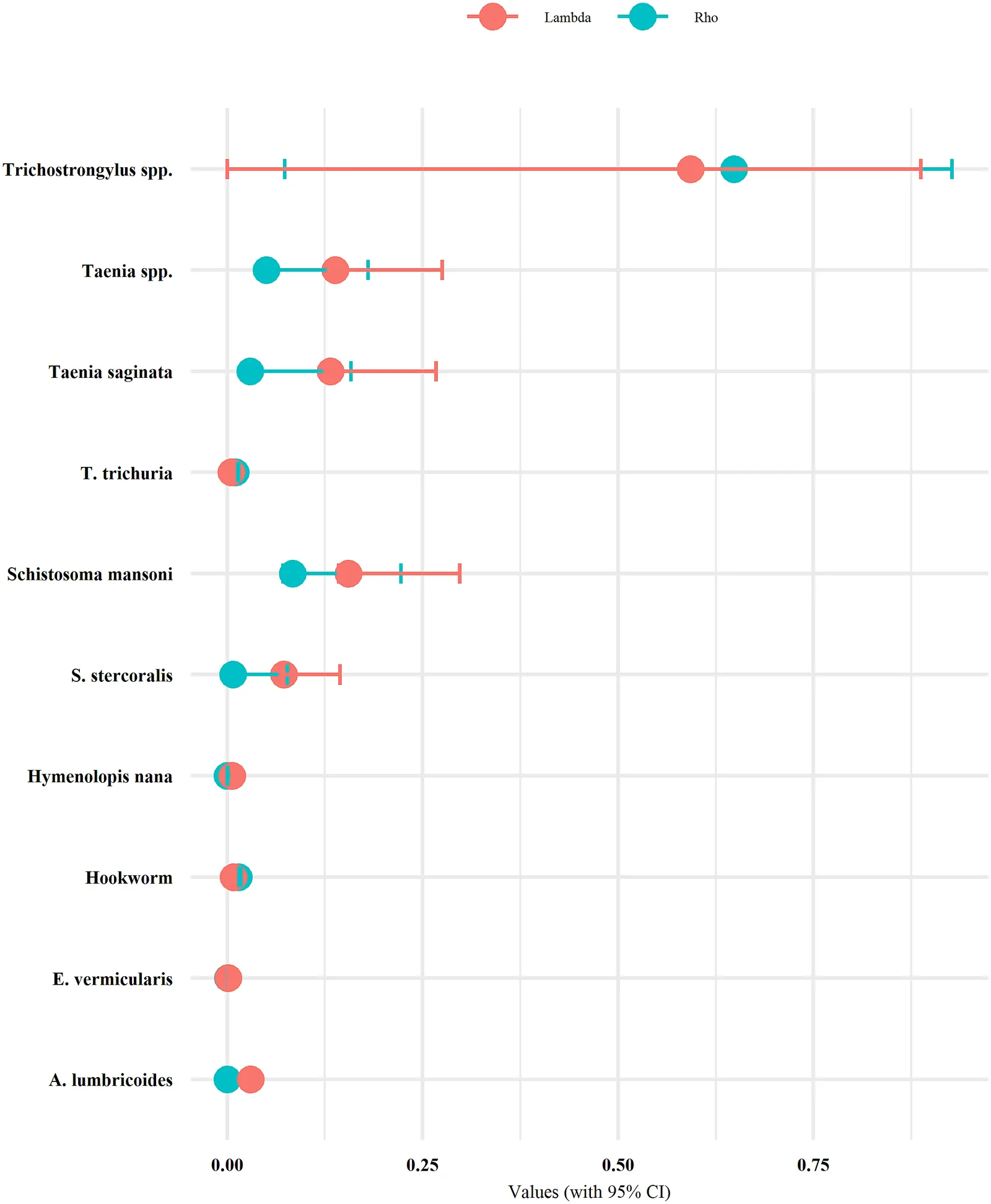
Estimated values (circles) and 95% Confidence Intervals (error bars) for the Force of Infection (FoI) and Infection Clearance Rate (ICR) for Different Species of Intestinal Helminthic Parasites among Food Handlers.

### 3.3 FoI and ICR by demographic, geographic, and methodological factors

By sex, males exhibited higher estimates than females. Specifically, FoI and ICR among males were 0.0394 (95% CI: 0.0375–0.0414) and 0.0352 (95% CI: 0.0345–0.0358), respectively, compared to 0.0149 (95% CI: 0.0125–0.0161) and 0.0175 (95% CI: 0.0161–0.0182) among females. By age, the highest values were observed in individuals aged 26–30 years, with FoI estimated at 0.0966 (95% CI: 0.0894–0.1652) and ICR at 0.1330 (95% CI: 0.1270–0.1724). By HDI, countries with low HDI levels showed lower infection dynamics (FoI: 0.0584, 95% CI: 0.0570–0.0684; ICR: 0.0760, 95% CI: 0.0752–0.0814), whereas countries with high HDI exhibited the highest values (FoI: 0.2070, 95% CI: 0.1694–0.2952; ICR: 0.2238, 95% CI: 0.2062–0.2437). By educational level, the highest estimates were found among illiterate individuals, with FoI at 0.0521 (95% CI: 0.0515–0.0559) and ICR at 0.0618 (95% CI: 0.0615–0.0638). By sample source, stool samples alone yielded a FoI of 0.0102 (95% CI: 0.0101–0.0110) and an ICR of 0.0170 (95% CI: 0.0169–0.0177). In contrast, combined stool and fingernail samples produced higher estimates, with FoI at 0.0147 (95% CI: 0.0125–0.0298) and ICR at 0.0229 (95% CI: 0.0211–0.0356). By setting, the highest FoI and ICR were observed among food handlers working in shops and restaurants, with FoI at 0.4271 (95% CI: 0.3598–0.5708) and ICR at 0.3451 (95% CI: 0.2971–0.3589). By diagnostic methodology, studies employing direct smear techniques alone reported a FoI of 0.0552 (95% CI: 0.0529–0.0738) and an ICR of 0.0861 (95% CI: 0.0843–0.0988), indicating a declining trend as ICR exceeded FoI, suggesting that the detected cases were likely acute or treated infections. Conversely, studies employing direct smear combined with concentration methods, which provide higher sensitivity for detecting low-intensity infections, yielded substantially lower point estimates: a FoI of 0.00368 (95% CI: 0.00367–0.00428) and an ICR of 0.002 (95% CI: 0.001–0.0055), showing an increasing trend (Table 3 and Figs. 5 and 6). This reflects a slow, negligible increase in prevalence in absolute terms, indicative of a stable, chronic endemic state. The near-zero ICR suggests minimal natural clearance, creating a stalemate where continuous low-level transmission outweighs negligible recovery, maintaining a persistent infection burden.

**Fig. 5:**
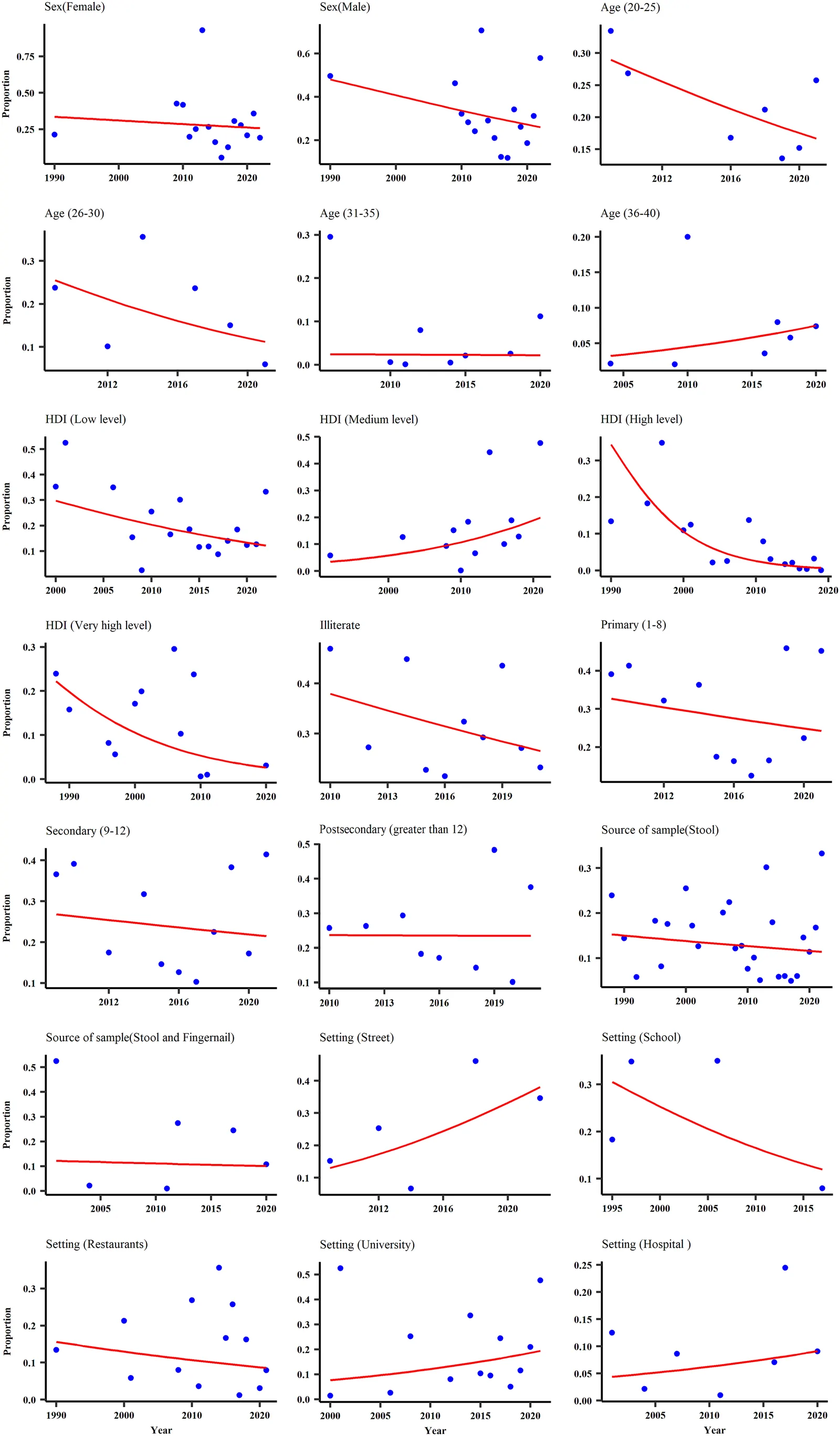

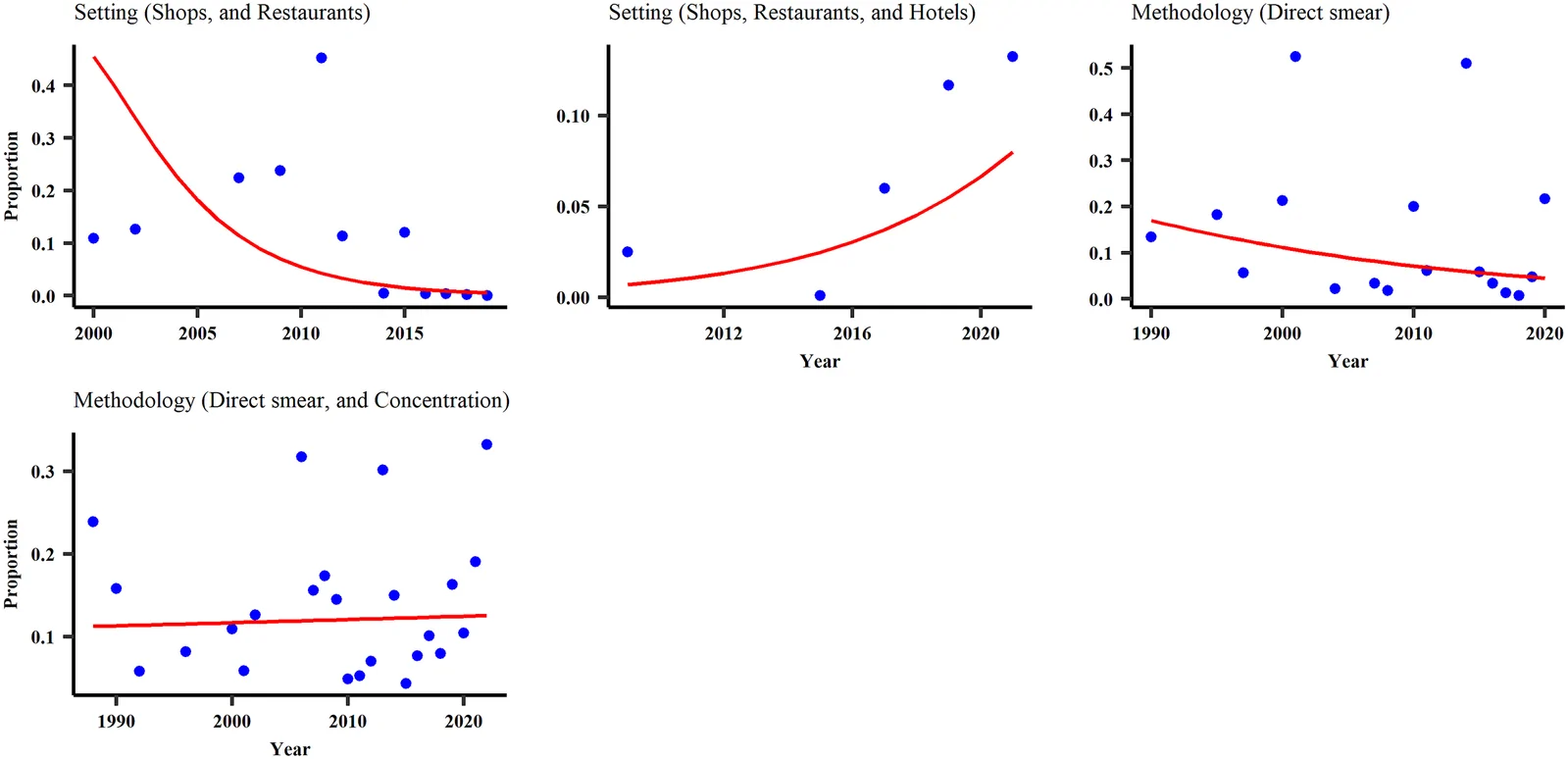
Force of Infection (FoI) and Infection Clearance Rate (ICR) for Intestinal Helminthic Infections among Food Handlers by Various Demographic, Geographic, and Methodological Factors, displaying observed values (blue dots) and fitted values (red lines). (For interpretation of the references to colour in this figure legend, the reader is referred to the web version of this article.)

**Fig. 6:**
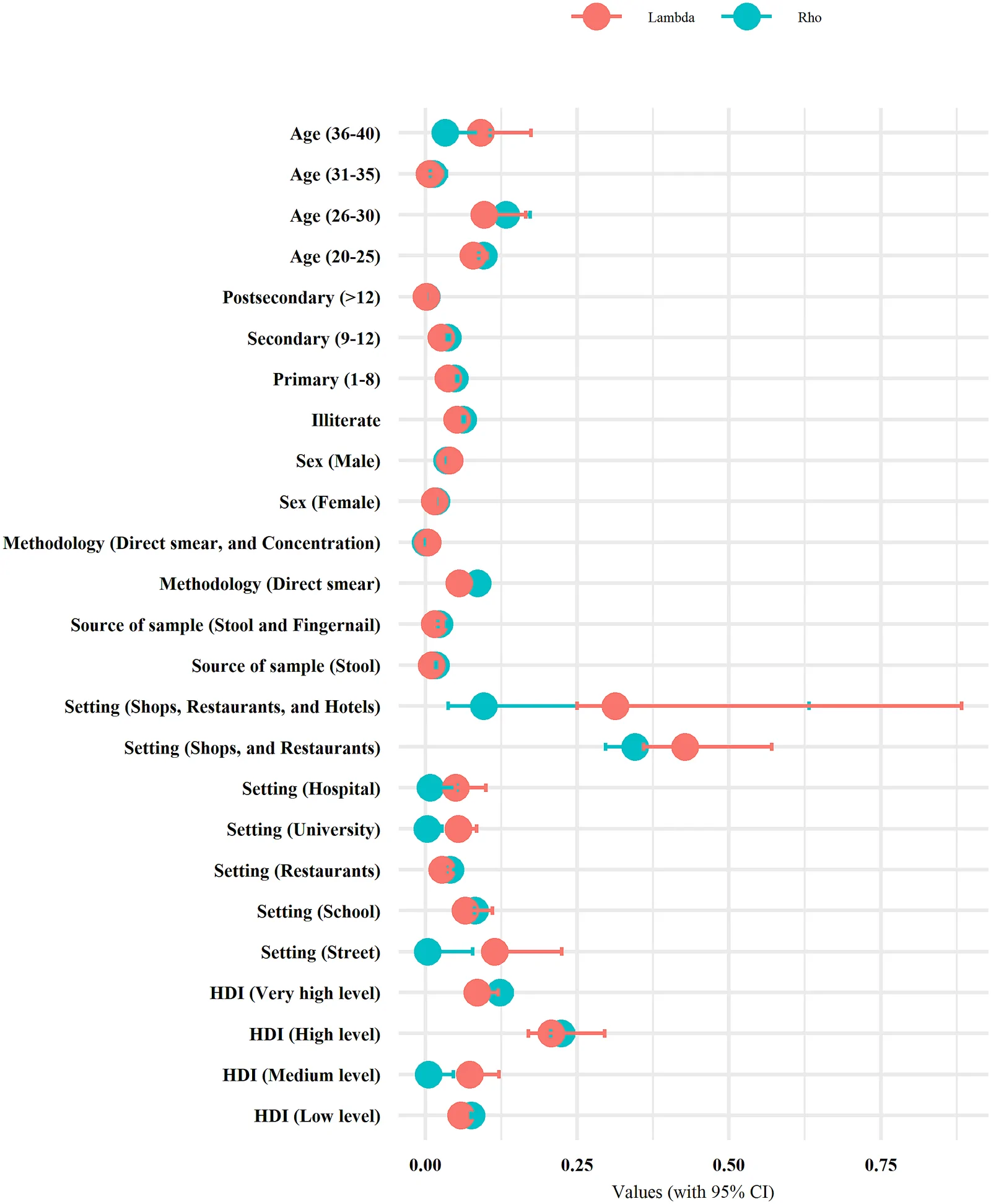
Estimated values (circles) and 95% Confidence Intervals (error bars) for the Force of Infection (FoI) and Infection Clearance Rate (ICR) for Intestinal Helminthic Infections among Food Handlers by Various Demographic, Geographic, and Methodological Factors.

Morover, Table 3 reveals that demographic and environmental subgroups exhibited wider RMSE ranges (0.0402–0.1969), reflecting higher heterogeneity. Specifically, environmental settings like shops/restaurants had lower RMSEs (0.0402–0.1096), while universities and combined settings showed higher variability (RMSE: 0.1706–0.1806). Diagnostic methods also varied, with concentration techniques yielding better fits (RMSE: 0.0855) than direct smear alone (RMSE: 0.1641).

**Table 3:** Force of Infection (FoI) and Infection Clearance Rate (ICR) with 95% Confidence Intervals (CI) and Root Mean Square Error (RMSE) for Intestinal Helminthic Infections among Food Handlers by Various Demographic, Geographic, and Methodological Factors⁎.

Groups	FoI	FoI Lower	FoI Upper	ICR	ICR Lower	ICR Upper	RSME
Sex							
Female	0.0149	0.0125	0.0161	0.0175	0.0161	0.0182	0.1969
Male	0.0394	0.0375	0.0414	0.0352	0.0345	0.0358	0.1547
Age							
20–25	0.0787	0.0652	0.1017	0.0959	0.0880	0.1086	0.0470
26–30	0.0966	0.0894	0.1652	0.1330	0.1270	0.1724	0.0935
31–35	0.0064	0.0000	0.0279	0.0136	0.0074	0.0346	0.1036
36–40	0.0908	0.0883	0.1743	0.0323	0.0299	0.1066	0.0607
HDI level							
Low	0.0584	0.0570	0.0684	0.0760	0.0752	0.0814	0.1040
Medium	0.0727	0.0669	0.1215	0.050	0.0001	0.0459	0.1298
High	0.2070	0.1694	0.2952	0.2238	0.2062	0.2437	0.0792
Very high	0.0849	0.0686	0.1201	0.1224	0.1123	0.1428	0.0972
Educational level							
Illiterate	0.0521	0.0515	0.0559	0.0618	0.0615	0.0638	0.0846
Primary (1–8)	0.0371	0.0368	0.0458	0.0483	0.0481	0.0535	0.1202
Secondary (9–12)	0.0255	0.0251	0.0301	0.0365	0.0362	0.0397	0.1137
Postsecondary (>12)	0.0011	0.0011	0.0014	0.0018	0.0018	0.0020	0.1152
Source of sample							
Stool	0.0102	0.0101	0.0110	0.0170	0.0169	0.0177	0.0788
Stool and Fingernail	0.0147	0.0125	0.0298	0.0229	0.0211	0.0356	0.1953
Setting							
Street	0.1143	0.1139	0.2247	0.037	0.034	0.0781	0.1081
School	0.0654	0.0653	0.1104	0.0812	0.0811	0.1009	0.1060
Restaurants	0.0274	0.0234	0.0411	0.0412	0.0381	0.0515	0.1096
University	0.0537	0.0521	0.0845	0.025	0.011	0.0272	0.1706
Hospital	0.0498	0.0486	0.0992	0.0080	0.0069	0.0519	0.0737
Shops, and Restaurants	0.4271	0.3598	0.5708	0.3451	0.2971	0.3589	0.1806
Shops, Restaurants, and Hotels	0.3123	0.2499	0.8831	0.0958	0.0373	0.6317	0.0402
Methodology							
Direct smear	0.0552	0.0529	0.0738	0.0861	0.0843	0.0988	0.1641
Direct smear, and Concentration	0.00368	0.00367	0.00428	0.002	0.001	0.0055	0.0855

⁎ Note: Values are rounded to four or five decimal places depending on the magnitude of the estimate to preserve the distinction between point estimates and confidence intervals.

## 4 Discussion

Our study provides the first global synthesis of FoI and ICR for intestinal helminths among food handlers, using a dynamic SIS model fitted to 32-year longitudinal prevalence data. The global burden shows a sustained decline, with clearance rates exceeding acquisition rates—likely reflecting improved sanitation, scaled-up mass drug administration (MDA), and stronger food safety regulations. This downward trend was broadly consistent across regions, most steeply in Africa, followed by Asia and South America, although India and Kenya exhibited expanding burdens requiring targeted surveillance. At the species level, only three of ten species demonstrated declining dynamics, with *Trichostrongylus* spp. showing the highest transmission parameters. Males, individuals aged 26–30 years, and Iran recorded the highest FoI estimates, though all within an overall declining pattern. Diagnostic method markedly influenced estimates, with concentration techniques detecting chronic infections that direct smear missed due to lower sensitivity.

The globally dynamics of FoI and ICR values in this study reflect a substantial decline in the burden of intestinal parasitic infections, with prevalence decreasing from an estimated 81.3% in the early 1990s to 23.8% during 2010–2011. This pronounced reduction is predominantly attributable to improvements in sanitation infrastructure, the widespread implementation of MDA programs, and the strengthening of food safety regulatory frameworks over the preceding three decades. (Alves de Oliveira Serra et al., 2015). Despite this overall progress, Asia and South America exhibit elevated transmission pressure compared to the global average. This elevated FoI is largely driven by regional climatic conditions that favor parasite survival, the continued agricultural use of untreated animal manure, and persistent hygiene gaps among food handlers (Bravo and Sanchez, 2003; Moreno, 2006). In response, these high-burden areas have implemented more intensive treatment and intervention efforts, which is reflected in their higher ICR. However, because their R₀ values remain elevated compared to the global value, their infection burden is declining at a slower pace. As a result, these regions sit closer to the equilibrium threshold, leaving them particularly vulnerable to a rapid resurgence in cases if current control measures are ever relaxed.

The high FoI and ICR observed for *Trichostrongylus* spp. can be attributed to their direct zoonotic lifecycle and environmental resilience, which facilitate rapid soil-to-host transmission, particularly in market and kitchen environments where animal manure is routinely handled (Mathison and Pritt, 2022). In contrast, *E. vermicularis* maintains low transmission and clearance rates (FoI and ICR), reflecting its predominantly household-clustered autoinfection cycle, which is readily disrupted by routine hand hygiene and laundering practices (Rawla and Sharma, 2026).

Furthermore, the observed FoI and ICR estimates for *E. vermicularis* in this food handler population indicate a low-transmission dynamic within this occupational setting. When interpreted jointly, these parameters yield an R₀ value consistent with stable low-prevalence trends, suggesting limited transmission pressure and reduced parasite circulation over time. This pattern likely reflects the mitigating effects of occupational hygiene practices and regulatory compliance inherent to food handler populations (Jothi et al., 2022).

Higher FoI and ICR in males may reflect differential occupational exposure patterns in food-handling settings. Emerging evidence suggests that men and women often undertake distinct tasks with varying exposure risks (Biswas et al., 2024; Putri and Susanna, 2021). However, direct quantitative data on gender-specific task distribution among food handlers are limited, and this interpretation remains tentative pending further occupational studies.

Age-stratified analysis revealed heterogeneous transmission dynamics across age groups. The 26–30 age group showed the highest FoI and ICR, likely reflecting high occupational exposure in early career stages factors (Balarak et al., 2016; Tefera and Mebrie, 2014). Despite this, R₀ = 0.73 indicates a net declining trend, suggesting adequate clearance mechanisms. In contrast, the 36–40 age group showed an expanding infection burden. This may reflect sustained exposure with inadequate clearance due to reduced health monitoring, accumulated chronic infections, or complacency in hygiene practices over longer employment tenure.

Interestingly, elevated FoI and ICR were observed across all HDI strata, challenging the assumption that higher HDI consistently reduces transmission. Specifically, low-HDI countries showed declining trends (likely due to underreporting and limited surveillance), medium-HDI countries exhibited increasing trends (reflecting recently improved monitoring that now captures previously hidden burden), and high-HDI countries showed declining trends (consistent with established control programs). The high-HDI paradox may be explained by the persistence of localized microenvironments—such as informal food sectors, street food vending, and densely populated markets—where suboptimal hygiene and favorable conditions for parasite transmission can exist despite overall national development (Javanmard et al., 2018, Kaminsky and Mäser, 2025). However, because our study did not directly assess such micro-environmental factors, this interpretation remains speculative and requires further investigation.

Sample type also influenced parameter estimates, such that combined stool and fingernail sampling yielded higher FoI and ICR values compared to stool alone, likely due to the retention of parasite eggs in periungual debris that are undetectable by fecal examination alone (Mengist et al., 2018; Sahlemariam and Mekete, 2001).

Diagnostic sensitivity critically shapes inferred transmission dynamics. Direct smear microscopy, being less sensitive, detects acute, high-intensity infections that are often treated, resulting in a declining trend (ICR > FoI). In contrast, Smear + Concentration, the gold standard for chronic infections, reveals a near-zero ICR (Alqarni et al., 2022; Oguoma and Ekwunife, 2007). This indicates a stable, chronic endemic state where continuous low-level transmission balances negligible natural clearance, highlighting the challenge of estimating dynamic clearance from stable, low-prevalence data.

Given the elevated FoI and ICR observed in specific populations and settings, targeted public health interventions are essential to interrupt transmission and reduce infection burden among food handlers. In hyperendemic regions, biannual MDA with broad-spectrum anthelmintics should be implemented for food handlers, alongside periodic parasitological screening to sustain control efforts (Alqarni et al., 2022; Yeshanew et al., 2021). Interrupting zoonotic transmission cycles of geohelminths requires a multifaceted approach, including the promotion of safe animal manure handling, enforcement of vegetable washing protocols, and improvement of sanitation in markets and food preparation areas (Mathison and Pritt, 2022). Regular food safety and personal hygiene training, proper hand-washing, nail trimming, and the use of protective equipment should be mandated for all food handlers, with the provision of literacy-appropriate materials tailored to those with limited formal education (Eshetu et al., 2019). Diagnostic protocols should prioritize concentration-based techniques and incorporate multi-site sample collection (e.g., stool and fingernail) to enhance diagnostic sensitivity and enable accurate monitoring of FoI and ICR trends, thereby supporting timely evaluation of intervention effectiveness (Soares et al., 2020). Biannual health certification for food handlers, including mandatory parasitological screening and treatment, should be enforced to reduce asymptomatic carriage and mitigate foodborne transmission risk (Kamau et al., 2012). Routine and rigorous health inspections have been shown to significantly reduce major food safety violations and improve hygiene compliance in food service establishments (Chatzimpyrou et al., 2025), with evidence suggesting that increased inspection frequency alone is associated with substantial gains in compliance indicators (Newbold et al., 2008). Educational attainment was a significant predictor of infection risk: illiterate food handlers exhibited a 7.37-fold increased risk compared to those with college education, while individuals with secondary education had a 5.97-fold elevated risk. Interestingly, food handlers with only primary education demonstrated lower infection risk than both illiterate and secondary-educated counterparts, warranting further investigation into this unexpected pattern (Getie et al., 2024). Finally, targeted food safety training programs have been shown to improve food handlers' knowledge, attitudes, and practices, forming a critical component of sustainable infection prevention strategies (Halim-Lim et al., 2023).

While this study provides novel insights into the transmission dynamics of intestinal helminths among food handlers through FoI and ICR modeling, several limitations warrant acknowledgment. First, the analysis relies on aggregated prevalence data derived from a published meta-analysis (Eslahi et al., 2023) rather than individual-level longitudinal data, which represents the gold standard for transmission modeling. To address this constraint, annualized estimates spanning 32 years (1990–2022) were employed as calibration targets for the dynamic SIS model, enabling inference of temporal trends in transmission and clearance rates. Nevertheless, the resulting FoI and ICR estimates remain model-dependent and may be influenced by temporal changes in diagnostic sensitivity, population demographics, or other unmeasured factors. Second, subgroups with fewer than three annual prevalence measurements were excluded to ensure reliable parameter estimation, eliminating 20 of 55 initial subgroups (36%) and limiting generalizability to regions or demographics with sparse temporal data. Sensitivity analyses for alternative inclusion thresholds were not performed; consequently, results may partially reflect the chosen criterion. Furthermore, subgroups with limited time points (e.g., Kenya with four measurements) exhibited wide confidence intervals, indicating substantial uncertainty. Third, as reported by the source meta-analysis, significant publication bias and considerable heterogeneity in diagnostic methods, sample collection protocols, and study populations may influence parameter estimates and limit comparability across studies. Despite these constraints, consistent patterns observed across geographic, demographic, and methodological strata reinforce the robustness of the findings. All estimates represent subgroup averages and should not be interpreted as individual-level risks to avoid ecological fallacy. These limitations notwithstanding, the findings underscore the utility of dynamic transmission models for informing public health surveillance. Future studies should prioritize longitudinal datasets and molecular diagnostics to refine FoI and ICR estimates, enabling more precise characterization of temporal trends and reinfection dynamics. Integration of such data into routine surveillance systems would facilitate early detection of transmission hotspots and support evidence-based targeting of intervention strategies.

Our study provides a strategic foundation for advancing global intestinal helminth control among food handlers. We propose a roadmap comprising: (1) enhanced surveillance through longitudinal monitoring and improved diagnostic protocols; (2) targeted interventions using subgroup-specific FoI and ICR data to direct MDA and hygiene promotion based on region and risk factors; (3) structural reforms including mandatory health certifications and regulatory oversight; and (4) future research on reinfection dynamics, One Health transmission, and mathematical modeling of hotspots. This approach prioritizes immediate diagnostic improvements, followed by adaptive treatment and policy enforcement, to sustainably reduce transmission.

## 5 Conclusion

This study presents the first global estimates of FoI and ICR for intestinal helminths among food handlers, derived from a dynamic SIS model calibrated to 32-year time-series prevalence data. The findings reveal significant heterogeneity across regions, species, and subpopulations, with R₀ below 1 in most settings, indicating a net declining trend in global infection burden. However, two notable exceptions—India and Kenya—exhibit expanding burdens (R₀ > 2), and most parasite species (7 of 10) show increasing transmission dynamics. These estimates are based on aggregated prevalence data and should be interpreted with caution, particularly for subgroups with limited temporal data. The results highlight the need for targeted interventions, including mass drug administration, hygiene training, enhanced diagnostics (particularly concentration-based methods), and regulatory enforcement, particularly in strata with elevated transmission pressure such as Asia and South America.

## Declaration of generative AI in scientific writing

We acknowledge the use of DeepSeek (version 2.3), an AI-based tool, for editing and improving the manuscript to enhance clarity and coherence. All modifications were reviewed and approved by the authors.

## Ethic Statment

This study was ethically approved by the Ethics Committee of Qom University of Medical Sciences, Iran (NO: IR.MUQ.REC.1404.063).

## CRediT authorship contribution statement

**Behrooz Ahmadi:** Writing – review & editing, Writing – original draft, Investigation, Data curation, Conceptualization. **Meysam Olfatifar:** Writing – review & editing, Visualization, Software, Project administration, Methodology, Funding acquisition, Formal analysis. **Milad Badri:** Writing – review & editing, Validation, Project administration, Conceptualization. **Alireza Ghorbani:** Writing – review & editing, Visualization. **Mahdi Fakhar:** Writing – review & editing, Visualization.

## Consent to participate

Not applicable.

## Consent for publication

Not applicable.

## Funding information

We express our gratitude to Qom University of Medical Sciences for their financial support of our project (Project code: 3822).

## Declaration of competing interest

The authors declare the following financial interests/personal relationships which may be considered as potential competing interests: Meysam olfatifar reports financial support was provided by Qom University of Medical Sciences. If there are other authors, they declare that they have no known competing financial interests or personal relationships that could have appeared to influence the work reported in this paper.

## Data Availability

All data used in this study are available in a recent meta-analysis accessible at https://doi.org/10.1016/j.foodcont.2023.109658.
